# Association of pre-chemotherapy peripheral blood pro-inflammatory and coagulation factors with reduced relative dose intensity in women with breast cancer

**DOI:** 10.1186/s13058-017-0895-5

**Published:** 2017-08-29

**Authors:** Yuan Yuan, Nilesh Vora, Can-Lan Sun, Daneng Li, Enrique Soto-Perez-de-Celis, Joanne Mortimer, The-hang Luu, George Somlo, James Waisman, David Smith, Joseph Chao, Vani Katheria, Timothy Synold, Vivi Tran, Shu Mi, Abrahm Levi, Anait Arsenyan, Jennifer Choi, Laura Zavala, Susan Yost, Arti Hurria

**Affiliations:** 1 City of Hope Comprehensive Cancer Center and Beckman Research Institute, Duarte, CA USA; 2 Long Beach Memorial Medical Center, Long Beach, CA USA

**Keywords:** Breast Cancer, Older adults, Aging, Biomarkers, Activities of Daily Living, Blood coagulation factors, Cytokines, Dose-response relationship, Drug, Chemotherapy, Adjuvant, Chemotherapy, Neoadjuvant

## Abstract

**Background:**

Chemotherapy decreases the risk of relapse and mortality in early-stage breast cancer (BC), but it comes with the risk of toxicity. Chemotherapy efficacy depends on relative dose intensity (RDI), and an RDI < 85% is associated with worse overall survival. The pro-inflammatory (interleukin (IL)-6, C-reactive protein (CRP)) and coagulation factors (D-dimer) serve as biomarkers of aging. The purpose of this study is to determine if these biomarkers are associated with reduced RDI in women with stage I–III BC.

**Methods:**

This study enrolled women with stage I–III BC. Prior to adjuvant or neoadjuvant chemotherapy, peripheral blood was collected for biomarker measurement. Dose reductions and delays were captured and utilized to calculate the RDI delivered. Univariate and multivariate analyses were performed to describe the association between pre-chemotherapy IL-6, CRP, and D-dimer levels and an RDI < 85%, controlling for relevant tumor and patient factors (age, stage, receptor status, chemotherapy regimen, and pre-chemotherapy physical function and comorbidity).

**Results:**

A total of 159 patients (mean age 58 years, range 30–81, SD 11.3) with stage I–III BC were enrolled. An RDI < 85% occurred in 22.6% (N = 36) of patients and was associated with higher pre-chemotherapy IL-6 (OR 1.14, 95% CI 1.04–1.25; *p* = 0.006) and D-dimer (OR 2.32, 95% CI 1.27–4.24; *p* = 0.006) levels, increased age (*p* = 0.001), increased number of comorbidities (*p* = 0.01), and decreased physical function by the Medical Outcomes Survey Activities of Daily Living (ADL) Scale (*p* = 0.009) in univariate analysis. A multivariate model, including two biomarkers (IL-6 and D-dimer), age, ADL, BC stage, and chemotherapy regimen, demonstrated a significant association between the increased biomarkers and reduced RDI < 85% (OR 2.54; *p* = 0.04).

**Conclusions:**

Increased pre-chemotherapy biomarkers of aging (IL-6 and D-dimer) are associated with reduced RDI (<85%). Future studies are underway to validate these findings.

**Trial registration:**

ClinicalTrials.gov, NCT01030250. Registered on 3 November 2016.

**Electronic supplementary material:**

The online version of this article (doi:10.1186/s13058-017-0895-5) contains supplementary material, which is available to authorized users.

## Background

The efficacy of chemotherapy is dependent on relative dose intensity, which is defined as the ratio of standard or planned dose intensity (dosage of drug delivered per unit of time) to actual dose intensity received. A reduction in the relative dose intensity (RDI) of adjuvant chemotherapy is associated with a loss of clinical benefit in women with breast cancer [1–7]. In particular, an RDI < 85% is associated with worse overall survival and increased age is noted to be a risk factor [1].

Although older adults are at increased risk for reduced RDI, chronological age alone is a poor predictor of biological age [8–11]. Biomarkers of aging could potentially be utilized to further understand the heterogeneity of the aging process. Growing evidence has demonstrated that pro-inflammatory and coagulation factors such as interleukin-6 (IL-6) [12–14], C-reactive protein (CRP) [12], and D-dimer may serve as potential biomarkers of aging. Elevated levels of these factors have been associated with reduced physical performance [15], frailty [16], and an increased risk of death [17] in community-dwelling older adults. The utility of these markers as predictors of chemotherapy tolerance, and the ability to deliver chemotherapy (i.e. RDI) in patients with breast cancer is unknown.

The goal of this study was to explore the association between biomarkers of aging (IL-6, CRP, and D-dimer) and the ability to deliver adjuvant or neoadjuvant chemotherapy. We hypothesized that elevated biomarkers of aging (and/or combinations of these biomarkers) may be associated with a reduced RDI among women with stage I–III breast cancer receiving adjuvant or neoadjuvant chemotherapy.

## Methods

This prospective longitudinal study was open at two participating institutions (City of Hope and Long Beach Memorial Medical Center). Eligible patients had a diagnosis of stage I–III breast cancer, were scheduled to receive adjuvant or neoadjuvant chemotherapy, and were fluent in English (since all measures in the patient questionnaires were not validated in other languages). Patients with stage IV disease were excluded. The study was approved by the institutional review boards (IRB) of the participating institutions (City of Hope IRB and Long Beach Memorial Medical Center IRB). Participating patients completed the informed consent process.

### Patient and tumor characteristics

Patient age, race/ethnicity, and socio-demographics were captured. Breast cancer stage, type of treatment, and receptor status (estrogen receptor (ER), progesterone receptor (PR), and human epidermal growth factor receptor 2 (HER2)) were collected.

### Biomarkers of aging

Prior to initiation of adjuvant or neoadjuvant chemotherapy, peripheral blood (7.5 ml) was collected for measurement of pro-inflammatory (IL-6 and CRP) and coagulation (D-dimer) factors. Plasma was stored at − 80 °C until assays were run. Quantitative IL-6 and CRP levels were measured using NOVEX® immunoassay (Invitrogen) and D-dimer levels were measured with Nanopia® D-dimer (Sekisui). Information regarding the time from surgery to peripheral blood collection was recorded for each of the patients who received chemotherapy in the adjuvant setting.

### Relative dose intensity and chemotherapy toxicities

The chemotherapy regimen was prescribed at the treating physician’s discretion. The chemotherapy dose (planned and received) and the reasons for dose reductions and dose delays were captured. RDI was calculated as the ratio of actual dose intensity received to the standard or planned dose intensity (dosage of drug delivered per unit of time). Trastuzumab dosing was not included in RDI calculations. Grade ≥ 3 toxicities defined by National Cancer Institute Common Terminology Criteria for Adverse Events (NCI CTCAE) Version 4.0 were captured. Emergency visits and hospitalizations were collected.

### Measures of physical function and comorbidity

All patients completed a questionnaire, which included measures of physical function and comorbidity. Measures of physical function included Activities of Daily Living (ADL) [18], Instrumental Activities of Daily Living (IADL) [19], Karnofsky Performance Status (KPS) [20], and Timed Up and Go (TUG) [21]. The ADL measured by the Medical Outcome Study (MOS) Physical Heath scale evaluated a range of activities including the ability to bathe independently and the ability to run, rated on a scale of 0–100, with a higher score indicating better physical function. The Instrumental Activities of Daily Living (IADL) scale measured the ability to complete activities that are required for independence in the community such as shopping and taking transportation, resulting in a score of 0–14, with a higher score signifying greater independence. The physician-rated KPS is a global measure of performance status measured on a scale of 0–100 with a higher score reflecting better function. Patient self-rated KPS is a global measure of performance status, which the patient self-rates from a scale of 40–100. The TUG is a performance-based measure of functional status that measures the time that it takes (in seconds) to rise from a chair, walk three feet, turn around, walk back to the chair and sit down.

Comorbidity was evaluated using the Older Americans Resources and Services Program (OARS) questionnaire [22] in which patients report if they have comorbidities such as hypertension, arthritis, diabetes mellitus, heart disease, and stroke. Each of these comorbidities has been associated with increased levels of IL-6, D-dimer, and/or CRP [12, 23–25].

### Statistical analyses

Descriptive analyses were performed to summarize patient, tumor, and treatment characteristics. The mean (SD) and median (inter-quartile) values were calculated for peripheral blood biomarkers (IL-6, CRP, and D-dimer) and physical function measures (ADL, IADL, TUG, patient self-rated KPS, and physician-rated KPS). The main outcome, RDI, was dichotomized using 85% as the cutoff point, with reduced RDI defined as RDI < 85%.

The Pearson product-moment correlation coefficient was computed to assess the relationship among the peripheral blood biomarkers. Logistic regression was used to examine the relationships between reduced RDI (< 85%) and peripheral blood biomarker (IL-6, CRP, and D-dimer) measurements. In the analyses, values of CRP, IL-6, and D-dimer were categorized using the 3^rd^ quartile as cutoff points (4^th^ quartile vs. quartiles 1–3) [17]. An IL-6 and D-dimer combination biomarker variable was created indicating 0 (reference group) if neither of the biomarker values were in the 4^th^ quartile, vs. 1 or 2 if one or two of the biomarkers were in the highest quartiles. In addition, we also examined the relationship between RDI < 85% and the following variables: age, number of comorbidities, body surface area (BSA), breast cancer stage, receptor status, chemotherapy regimen, adjuvant vs. neoadjuvant chemotherapy, time from surgery to peripheral blood collection (in patients treated in the adjuvant setting), and physical function measures (ADL, IADL, TUG, patient self-rated KPS and physician-rated KPS). Age and cancer stage were kept in the multivariate model regardless of their *p* values. For other variables, stepwise selection was used to select the final variables to be included in the model. All statistical tests were two-sided, and *p* values <0.05 were considered statistically significant. Data were analyzed using SAS 9.3 (SAS Institute, Cary, NC, USA).

## Results

### Patient, tumor, and treatment characteristics

Between July 2009 and December 2014, 206 patients with stage I–III breast cancer were accrued to this study. The current analysis included 159 patients who consented for the pre-chemotherapy blood biomarker measurements. There were no significant differences between the 159 patients who contributed and the 46 patients who declined blood sample, in terms of age, race/ethnicity, disease stage, hormone receptor status, and functional status. However, patients who declined participation had increased comorbidities (*p* < 0.01).

One hundred fifty nine patients (median age 59 years, range 30–81) with stage I–III breast cancer (stages I (21%), II (55%), III (24%)) were included in this analysis. Among them, 52% (N = 82) were under the age of 60; 29% (N = 46) were 60–69, and 19% (N = 31) were 70 years of age and older. Among the patients, 50% were non-Hispanic white, 25% were Hispanic, 11% were African American, and 9% were Asian.

The percentage of the population with ER-positive HER2-negative disease was 67% (N = 106), 18% (N = 28) of the population were triple negative, and 16% (N = 25) were HER2-positive: 87% of patients (N = 142) received adjuvant chemotherapy and 47% (N = 75) received non-anthracycline-containing regimens (Table 1). Grade 3–5 chemotherapy toxicities were experienced by 44% of patients (N = 70): 13 patients experienced hematologic toxicity alone, 38 experienced non-hematologic toxicities alone, and 19 had both hematologic and non-hematologic toxicities. The most frequently encountered toxicities are summarized in Table 2. Among the 159 patients, 31 patients were hospitalized and 46 had an emergency department visit during chemotherapy.

**Table 1:** Patient and treatment characteristics (N = 159 patients)

Baseline characteristics		
Age, years	59.0	30–81
Age group, years		
< 50	38	23.9%
50 to < 60	44	27.5%
60 to < 70	46	28.8%
≥ 70	31	19.4%
Race		
Non-Hispanic white	80	50.3%
Hispanic	40	25.2%
African-American	17	10.7%
Asian	15	9.4%
Others	6	3.8%
Missing data	1	0.6%
Breast cancer stage		
I	34	21.4%
II	88	55.3%
III	37	23.3%
ER, PR, HER2 status		
ER+ or PR+, HER2-	106	66.7%
HER2+	25	15.7%
ER-PR-HER2-	28	17.6%
Type of chemotherapy		
Neoadjuvant	17	10.7%
Adjuvant	142	89.3%
Chemotherapy regimen		
AC-T	56	35.2%
TC	59	37.1%
AC-TH	11	6.9%
TCH	11	6.9%
Sequential A-T-C	10	6.3%
Other non-HER2-targeted therapy	6	3.8%
Other HER-2-targeted therapy	6	3.8%
Anthracycline-containing regimen		
No	75	47.2%
Yes	84	52.8%
Comorbidities		
Hypertension	54	34.0%
Arthritis	47	29.6%
Depression	31	19.5%
Circulation problem	22	13.8%
Other cancers	18	11.3%
Stomach disorders	17	10.7%
Other^a^		
Number of comorbidities ≥ 1	105	66.0%
Number of comorbidities ≥ 2	64	40.3%
Number of comorbidities ≥ 3	35	22.0%

Values are presented as median and range, or number and percentage

*ER* estrogen receptor, *PR* progesterone receptor, *HER2* human epidermal growth factor receptor 2, *TC* docetaxel plus cyclophosphamide, *AC-T* doxorubicin plus cyclophosphamide followed by paclitaxel, *TCH* docetaxel, carboplatin and trastuzumab, *AC-TH* doxorubicin plus cyclophosphamide followed by paclitaxel plus trastuzumab, *A-T-C* sequential doxorubicin, paclitaxel and cyclophosphamide

^a^Other: heart disease (N = 15, 9.4%); diabetes mellitus (N =12, 7.5%); glaucoma (N = 9, 5.7%); emphysema (N = 5, 3.1%); liver/kidney disease (N = 2, 1.3%); stroke (N = 1, 0.6%)

**Table 2:** Type of toxicity among patients with treatment-related grade 3–5 toxicities (N = 70 patients)

Toxicity type	Grade 3–4 toxicities	
Hematologic		
Anemia	13	38%
White blood cell count decreased	10	29%
Neutrophil count decreased	8	24%
Bleeding	3	9%
Non-hematologic		
Metabolic abnormalities	14	12%
Nausea/vomiting	13	11%
Neuropathy	12	10%
Mucositis	9	8%
Infection	9	8%
Fatigue	9	8%
Diarrhea	6	5%
Arrhythmia	5	4%
Left ventricular systolic dysfunction	5	4%
Pain	5	4%
Dehydration	4	3%
Allergy	3	2.5%
Hypotension	3	2.5%
Syncope	3	2.5%
Kidney dysfunction	3	2.5%
Dizziness	2	1.7%
Others^a^	1	0.8%

Results are presented as number and percentage of patients

^a^Others: depression, falls, dysphagia, abnormal liver enzyme, bowel perforation, constipation, sore throat, aspiration, embolism, hand-foot syndrome

### Measures of physical function and comorbidity

The physical function measures are summarized in Table 3. Physician-rated KPS ranged from 80 to 100, with > 95% of patients being rated ≥ 90. The patient self-rated KPS ranged from 50 to 100, with > 85% of patients rating their KPS ≥ 80. Median MOS Physical Health (ADL) score was 90 (range 0–100). The median IADL score was 14 (range 4–14). Thirty patients (21%) reported at least one fall in the last 6 months. The median score on the TUG was 9.5 seconds (range 5–18). There were 66% of patients (N = 105) with one or more comorbidities, and 22% (N = 35) reported three or more comorbidities.

**Table 3:** Measures of physical function

Physical function	Mean	SD	Median	Range	Q1, Q3
Patient self-rated KPS	89.8	13.24	90	50–100	90, 100
Physician-rated KPS	94.7	5.85	100	80–100	90, 100
ADL (MOS physical health)	79.4	22.64	90	0–100	70, 95
IADL (subscale of OARS)	12.3	2.86	14	4–14	12, 14
TUG	9.7	2.22	9.5	5.1–17.9	8.2, 10.8

*KPS* Karnofsky Performance Status, *ADL* Activities of Daily Living, *MOS* Medical Outcome Study Physical Heath Scale, *IADL* Instrumental Activities of Daily Living, *OARS* Older Americans Resources and Services Program, TUG Timed Up and Go, *Q* Quartile

### Peripheral blood biomarkers of aging

The median time from surgery to peripheral blood sample collection in patients treated with adjuvant chemotherapy was 52 days (range 18–193 days). The mean and median values of the three biomarkers in peripheral blood are shown in Table 4. There was modest positive correlation between IL-6 and CRP levels (*r* = 0.37; *p* < 0.001). No correlation was found between D-dimer levels and either IL-6 (*r* = 0.12; *p* = 0.12) or CRP levels (*r* = 0.07; *p* = 0.35). No significant changes in the mean biomarker levels were found when patients who received neoadjuvant chemotherapy were excluded from the analysis (Additional file 1: Table S1).

**Table 4:** Measures of peripheral blood biomarkers

Biomarkers	Mean	SD	Median	Range	Q1, Q3
D-dimer (μg/ml)	0.8	0.59	0.6	0.1–3.3	0.4, 1.1
IL-6 (pg/ml)	3.4	4.82	1.9	0–42.1	0.3, 5.0
CRP (μg/ml)	5.5	7.81	2.8	0.1–48.4	1.4, 6.0

*CRP* C-reactive protein, *Q* Quartile

### RDI

The median RDI was 90% (range 20–100%), with 23% of patients (N = 36) receiving an RDI < 85%. Patients who experienced grade 3–5 toxicities were more likely to receive an RDI < 85% (40% vs. 9%; *p* < 0.001). Compared to patients who never experienced grade 3–5 toxicities, those experiencing grade 3–5 non-hematological toxicities were significantly more likely to receive an RDI < 85% (OR 8.49, 95% CI 3.47–20.76; *p* < 0.001), while those who experienced hematological toxicities alone were not (OR 1.84, 95% CI 0.35–9.81; *p* = 0.47). Patients who were hospitalized during chemotherapy were more likely to receive an RDI < 85% than those who were not hospitalized (55 vs. 15%; *p* < 0.001). Patients with an emergency department visit during chemotherapy were as likely to receive an RDI < 85% as those without emergency department visits (24% vs. 22%; *p* = 0.81).

In univariate analysis, older age (*p* = 0.001), increased number of comorbidities (*p* = 0.01), and reduced physical function as measured by the ADL scale (*p* = 0.009) were associated with an RDI < 85% (Table 5). In patients treated with adjuvant chemotherapy, the time from surgery to peripheral blood draw was not significantly associated with either biomarker levels or with an RDI < 85%. The various chemotherapy regimens used, and their association with an RDI < 85%, are also shown in Table 5. Increased IL-6 (OR 1.14, 95% CI 1.04–1.25; *p* = 0.006) and D-dimer levels (OR 2.32, 95% CI 1.27–4.24; *p* = 0.006) were associated with an RDI < 85%. No association was found between increased CRP levels and an RDI < 85% (*p* = 0.47) analyzed as a continuous variable (Table 6). When examined as categorical variables, patients with IL-6 (OR 2.49, 95% CI 1.12–5.56; *p* = 0.02) and/or D-dimer (OR 3.52, 95% CI 1.6–7.78; *p* = 0.002) levels in the highest (4^th^) quartile were more likely to have an RDI < 85% compared to those with IL-6 and D-dimer levels in quartiles 1–3. This association was not found for CRP levels (OR 1.25, 95% CI 0.53–2.89; *p* = 0.61). When combining IL-6 and D-Dimer, those patients with one or more biomarkers in the 4^th^ quartile had an increased risk of an RDI < 85% (OR 3.59, 95% CI 1.64–8.87; *p* = 0.001). No significant changes in the association between biomarkers of aging and reduced RDI were found when patients who received neoadjuvant chemotherapy were excluded from the analysis (Additional file 2: Table S2).

**Table 5:** Univariate associations between demographic, clinical and GA variables and RDI < 85%

	RDI ≥ 85% (N = 123)	RDI < 85% (N = 36)	OR (95% CI)	*P* values
Continuous variables				
Age (per year)	56.7 (11.20)	64.2 (11.78)	1.06 (1.02–1.10)	0.001
BMI	29.3 (6.06)	30.4 (7.07)	1.03 (0.97–1.09)	0.363
BSA	1.6 (0.13)	1.6 (0.15)	1.00 (0.06– 5.72)	0.999
Number of comorbidities	1.3 (1.57)	2.3 (2.21)	1.30 (1.06–1.59)	0.01
ADL (MOS physical health)	82.0 (21.30)	70.4 (24.99)	0.98 (0.96–0.99)	0.009
IADL	12.4 (2.84)	11.9 (2.93)	0.95 (0.84–1.07)	0.407
TUG	9.7 (2.23)	9.9 (2.19)	1.04 (0.88–1.22)	0.681
Patient self-rated KPS	90.6 (12.30)	87.2 (15.97)	0.98 (0.96–1.01)	0.185
Physician-rated KPS	95.0 (5.34)	93.5 (7.34)	0.96 (0.90–1.02)	0.184
Number of falls				
0	98 (79.0%)	26 (21.0%)	1.00	
1+	23 (69.7%)	10 (30.3%)	1.64 (0.69–3.87)	0.26
Categorical variables				
Race				
Hispanic	33 (82.5%)	7 (17.5%)	1.00	
Non-Hispanic white	63 (78.8%)	17 (21.3%)	1.27 (0.48–3.38)	0.63
Asian	10 (66.7%)	5 (33.3%)	2.36 (0.61–9.08)	0.21
African-American	13 (76.5%)	4 (23.5%)	1.45 (0.36–5.80)	0.60
Others	3 (50.0%)	3 (50.0%)	4.71 (0.78–28.41)	0.09
Missing	1			
ER–PR, HER-2 status				
ER+ or PR+, HER2-	83 (78.3%)	23 (21.7%)	1.00	
HER2+	17 (68.0%)	8 (32.0%)	1.70 (0.65–4.43)	0.28
ER- PR- HER2-	23 (82.1%)	5 (17.9%)	0.7 8 (0.27–2.29)	0.66
Stage				
Stage I	27 (79.4%)	7 (20.6%)	1.00	
Stage II	65 (73.9%)	23 (26.1%)	1.36 (0.52–3.56)	0.52
Stage III	31 (83.8%)	6 (16.2%)	0.75 (0.22–2.49)	0.63
Chemotherapy regimen				
AC-T	46 (82.1%)	10 (17.9%)	1.00	
TC	51 (86.4%)	8 (13.6%)	0.72 (0.26–1.98)	0.53
AC-TH	7 (63.6%)	4 (36.4%)	2.63 (0.64–10.72)	0.18
TCH	8 (72.7%)	3 (27.3%)	1.73 (0.39–7.68)	0.47
Sequential A-T-C	4 (40%)	6 (60%)	6.90 (1.64–29.06)	0.009
Other non-HER2-targeted therapy	4 (66.7%)	2 (33.3%)	2.30 (0.37–14.34)	0.37
Other HER-2-targeted therapy	3 (50%)	3 (50%)	4.60 (0.81–26.22)	0.09
Anthracycline-containing regimen				
No	61 (81.3%)	14 (18.7%)	1.00	
Yes	62 (73.8%)	22 (26.2%)	1.55 (0.72–3.30)	0.26

Continuous variables are presented as mean (SD) and categorical variables as N (%)

*GA* geriatric assessment, *RDI* reduced dose intensity, *BSA* body surface area, *BMI* body mass index, *AD*L Activities of Daily Living, *MOS* Medical Outcome Study Physical Heath Scale, *IADL* Instrumental Activities of Daily Living, *TUG* Timed Up and Go, *KP*S Karnofsky Performance Status, *TC* docetaxel plus cyclophosphamide, *AC-T* doxorubicin plus cyclophosphamide followed by paclitaxel, *TCH* docetaxel, carboplatin and trastuzumab, *AC-TH* doxorubicin plus cyclophosphamide followed by paclitaxel plus trastuzumab, *A-T-C* sequential doxorubicin, paclitaxel and cyclophosphamide

**Table 6:** Univariate association between peripheral blood biomarkers of aging and reduced RDI

	RDI ≥ 85%	RDI < 85%	OR (95% CI)	*P* value
	(N = 123)	(N = 36)		
As continuous variables				
IL-6 (pg/ml)	2.7 (3.15)	5.7 (7.95)	1.14 (1.04–1.25)	0.006
D-dimer (μg/ml)	0.7 (0.58)	1.1 (0.57)	2.32 (1.27–4.24)	0.006
CRP (μg/ml)	5.3 (7.42)	6.4 (9.07)	1.02 (0.97–1.06)	0.47
As categorical variables				
IL-6				
Q 1, 2, 3	98 (79.7%)	22 (61.1%)		
Q4	25 (20.3%)	14 (38.9%)	2.49 (1.12–5.56)	0.02
D-Dimer				
Q 123	97 (78.9%)	18 (51.4%)		
Q4	26 (21.1%)	17 (48.6%)	3.52 (1.60–7.78)	0.002
CRP				
Q 123	94 (76.4%)	26 (72.2%)		
Q 4	29 (23.6%)	10 (27.8%)	1.25 (0.53–2.89)	0.61
IL-6 or D-dimer combination^a^				
0	79 (86.8%)	12 (13.2%)		
1 or 2	44(64.7%)	24 (35.3%)	3.59 (1.64–8.87)	0.001

Continuous variables are presented as mean (SD) and categorical variables as N (%)

*RDI* relative dose intensity, *CRP* C-reactive protein

^a^IL-6 or D-dimer combination: 0 = both markers in lower three quartiles; 1 = 1 of the markers in the 4^th^ quartile; 2 = 2 of the markers in the 4^th^ quartile

In multivariate analysis, the association between an IL-6 and D-dimer level in the highest (4^th^) quartile and an RDI < 85% remained significant (OR 2.54, 95% CI 1.03–6.23; *p* = 0.04) after adjusting for patient age, physical function (ADL score), breast cancer stage, and receipt of an anthracycline regimen (Table 7). Although the number of comorbidities was associated with an RDI < 85% in univariate analysis, it was no longer significant after stepwise selection, and thus was not selected to be included in the final model.

**Table 7:** Multivariate association between biomarkers and RDI < 85%

Biomarkers/Clinical factors	Odds ratio (95%CI)	*P* value
IL-6 or D-dimer combination^a^		
1 or 2 vs. 0	2.54 (1.03–6.23)	0.04
Age	1.06 (1.02–1.10)	<0.01
Physical function measured by MOS-ADL^b^	0.98 (0.96–1.00)	0.04
Breast cancer stage		
Stage II vs. I	0.66 (0.22–1.98)	0.46
Stage III vs. I	0.23 (0.05–0.97)	0.04
Anthracycline-containing regimen		

^a^ IL-6 or D-dimer combination: 0 = both markers in lower three quartiles; 1 = 1or 2 of the markers in the 4^th^ quartile

^b^MOS-ADL: Activities of Daily Living (subscale of MOS physical health)

## Discussion

This study demonstrates an association between elevated pre-chemotherapy peripheral blood biomarkers of aging (IL-6 and D-dimer) and reduced RDI in women with stage I–III breast cancer receiving neoadjuvant or adjuvant chemotherapy. These biomarkers could potentially be of utility in oncology practice to understand “biological age” and the ability to deliver chemotherapy.

RDI represents the proportion of the dose intensity actually delivered compared with the standard dose intensity for a chemotherapy regimen [26]. Dose reductions or delays in the administration of chemotherapy due to toxicity, or with the intention to avoid toxicity, lead to a reduction in the dose intensity of treatment, and may decrease its therapeutic effect. Maintenance of chemotherapy dose intensity is necessary to maintain chemotherapy efficacy. A reduction in RDI to < 85% has been shown to correlate with worse survival in women with both early and advanced-stage breast cancer [27, 28], and optimizing RDI may be a valuable strategy to improve outcomes. Nevertheless, RDI reductions are common in clinical practice, and a large nationwide study of community practices identified RDI reductions of 15% or more in over half of patients with early-stage breast cancer [29]. Across several studies, advanced age has consistently been found to be a risk factor for a decreased RDI, and older women with breast cancer are more likely to have a reduced RDI compared with younger women [2, 30–34].

Although advanced age is a known risk factor for reduced RDI, chronological age is often a poor reflection of the physiological and functional status of older adults, and new tools are needed to determine which older patients are at greater risk of toxicity and a reduced RDI. At a molecular level, aging involves complex processes such as oxidative stress, inflammation, DNA damage, shortening telomeres, genotoxicity and genomic changes [35, 36]. In an attempt to measure the aging process, several potential biomarkers have been evaluated both in community dwelling older adults and in older adults with cancer. Among these, some of the most widely studied biomarkers of aging are inflammatory mediators and coagulation factors, such as IL-6, TNF-α, CRP and D-dimer. Inflammatory and coagulation markers are easily measured, increase with age, and are associated with functional decline and increased mortality in community-dwelling older adults [8, 12, 13, 25, 37–41].

The aforementioned biomarkers have also been studied in the context of oncology patients; however, the data focus more on the association between these biomarkers at the time of diagnosis and disease characteristics and cancer outcomes rather than their utility as biomarkers of aging. For example, elevated pre-treatment serum IL-6 correlates with a more advanced cancer stage and poorer response to chemotherapy [40]. Elevated CRP levels at the time of diagnosis of breast cancer are associated with reduced overall and disease-free survival and an increased risk of death from breast cancer [24]. In addition, among untreated patients with operable breast cancer, elevated plasma D-dimer levels are associated with lymphovascular invasion, higher clinical stage, and lymph node involvement [41].

In contrast with our study, most previous publications have focused on changes in biomarkers of aging before and after adjuvant chemotherapy. One study demonstrated that expression of p16INK4a, an inhibitor of cyclin-dependent kinase 4 associated with cellular senescence, increased after adjuvant chemotherapy in 33 patients with breast cancer, and remained elevated 12 months after treatment [42]. In another study of 57 patients age 70 years and older with breast cancer undergoing chemotherapy, there were modest decreases in IL-10 and insulin-like growth factor-1 (IGF-1), and increases in TNF-α and monocyte chemotactic protein 1 (MCP-1) from baseline to 1 year, which were significantly more pronounced in the chemotherapy group compared to the control group, suggesting accelerated biological aging [43]. A recent study by Extermann et al. found that there were no differences in peripheral biomarkers of aging (IL-6, D-dimer, IGF-1, and TNF- α) between patients (N = 27) with breast cancer at 1–2 years after completion of adjuvant chemotherapy and non-chemotherapy controls (N = 29) [44]. While previous publications show chemotherapy may lead to accelerated aging, information on the relationship between biomarkers of aging and chemotherapy tolerance is lacking, and this study is the first to our knowledge to show an association between pre-chemotherapy biomarkers of aging and the ability to deliver chemotherapy successfully.

Limitations of this study include the modest sample size and the fact that the biomarker component was optional; however, more than 75% of patients participated in the biomarker component. Furthermore, while the majority of the studies of biomarkers of aging were conducted among older adults, in this study we included patients across the aging spectrum. This could be considered a limitation or a strength, as our results could potentially be applied to patients across the aging spectrum. Patients receiving both neoadjuvant and adjuvant chemotherapy were included in this study. However, only a small proportion of the patients were treated with neoadjuvant chemotherapy, and the results of our analyses were not modified by the exclusion of this group of patients.

This study is hypothesis generating, and our findings need to be validated in an independent cohort of patients before biomarkers of aging can be integrated into the clinical care of women with breast cancer. However, if these findings are confirmed, biomarkers of aging could potentially be utilized before starting chemotherapy, since they could help identify those patients who are less likely to successfully complete planned treatments. This, in turn, could lead to the selection of less toxic chemotherapy regimens for those patients, or more intensive follow up aimed at the earlier detection of dose-limiting toxicities. An ongoing multicenter study of older adults with breast cancer (N = 500) undergoing adjuvant chemotherapy is close to completing accrual, and will be able to verify our findings and provide information on which specific chemotherapy regimens are more likely to be successfully completed (ClinicalTrials.gov identifier NCT01472094).

## Conclusions

This study is novel in connecting the fields of geriatrics and oncology by demonstrating the potential utility of biomarkers of aging in patients with early-stage breast cancer across the aging spectrum who received chemotherapy. This finding expands our current understanding of the association between the biomarkers of aging and delivery of chemotherapy. Future studies are underway to verify these findings.

## Additional files

Additional file 1: Table S1. Biomarkers measurement in neo-adjuvant-treated vs. adjuvant-treated patients. (DOC 39 kb)

Additional file 2: Table S2. Biomarkers and RDI < 85% in overall patients vs. adjuvant patients. (DOC 31 kb)

## Abbreviations

ADL: Activities of daily living
BSA: Body surface area
CRP: C-reactive protein
ER: Estrogen receptor
HER2: Human epidermal growth factor receptor 2
IADL: Instrumental activities of daily living
IGF-1: Insulin-like growth factor-1
IL-10: Interleukin-10
IL-6: Interleukin
KPS: Karnofsky Performance Status
MCP-1: Monocyte chemotactic protein 1
MOS: Medical Outcome Study
OARS: Older Americans Resources and Services Program
PR: Progesterone receptor
RDI: Relative dose intensity
TNF-α: Tumor necrosis factor-α
TUG: Timed Up and Go
