# Supplementary material for: Association of pre-chemotherapy peripheral blood pro-inflammatory and coagulation factors with reduced relative dose intensity in women with breast cancer
Source: Breast Cancer Res. 2017 Aug 29;19:101. doi: 10.1186/s13058-017-0895-5 (PMC5576099; doi:10.1186/s13058-017-0895-5)
Supplement: Supplementary file 1 — Biomarkers measurement in neo-adjuvant-treated vs. adjuvant-treated patients. (DOC 39 kb) [file 13058_2017_895_MOESM1_ESM.doc]

**Additional file 1: Table S1. Biomarkers Measurement in Neo-adjuvant vs Adjuvant Patients**

|  | **Neo-adjuvant**  **Mean (SD)** | **Adjuvant**  **Mean (SD)** | **P** | **Neo-adjuvant**  **Median (IQR)** | **Adjuvant**  **Median (IQR)** | **P** |
| --- | --- | --- | --- | --- | --- | --- |
| Overall N | 17 | 142 |  | 17 | 142 |  |
| IL6 | 4.6 (3.67) | 3.3 (4.93) | 0.27 | 4.1 (2.5-5.9) | 1.7 (0.3-4.0) | 0.02 |
| CRP | 8.0 (8.71) | 5.2 (7.67) | 0.16 | 5.5 (1.2-12.1) | 2.7 (1.5-5.7) | 0.44 |
| D-dimer | 0.7 (0.35) | 0.8 (0.62) | 0.52 | 0.7 (0.4-0.9) | 0.6 (0.4-1.1) | 0.43 |
| RDI>=85%, N | 13 | 110 |  | 13 | 110 |  |
| IL6 | 3.5 (2.86) | 2.6 (3.18) | 0.32 | 3.9 (1.0-5.7) | 1.7 (0.2-3.7) | 0.12 |
| CRP | 7.7 (7.54) | 5.0 (7.39) | 0.22 | 5.5 (1.3-12.1) | 2.5 (1.3-5.7) | 0.22 |
| D-dimer | 0.7 (0.39) | 0.7 (0.60) | 1.00 | 0.7 (0.4-1.0) | 0.5 (0.3-1.0) | 0.43 |
| RDI<85% , N | 4 | 32 |  | 4 | 32 |  |
| IL6 | 8.1 (4.24) | 5.4 (8.29) | 0.54 | 7.6 (4.8-11.4) | 2.0 (0.5-8.5) | 0.11 |
| CRP | 9.2 (13.24) | 6.0 (8.65) | 0.52 | 3.9 (0.6-17.8) | 3.2 (2.2-6.2) | 0.84 |
| D-dimer | 0.7 (0.19) | 1.1 (0.58) | 0.14 | 0.6(0.5-0.8) | 1.1 (0.6-1.7) | 0.18 |
