# Supplementary material for: Association of pre-chemotherapy peripheral blood pro-inflammatory and coagulation factors with reduced relative dose intensity in women with breast cancer
Source: Breast Cancer Res. 2017 Aug 29;19:101. doi: 10.1186/s13058-017-0895-5 (PMC5576099; doi:10.1186/s13058-017-0895-5)
Supplement: Supplementary file 2 — Biomarkers and RDI < 85% in overall patients vs. adjuvant patients. (DOC 31 kb) [file 13058_2017_895_MOESM2_ESM.doc]

**Additional file 2: Table S2. Biomarkers and RDI < 85% in Overall vs Adjuvant Patients**

| **Biomarkers/Clinical factors**  **Univariate results** | **Overall** | | **Adjuvant only** | |
| --- | --- | --- | --- | --- |
|  | **Odds Ratio (95%CI)** | **p** | **Odds Ratio (95%CI)** | **p** |
| IL-6 (pg/ml) | 1.14 (1.04-1.25) | 0.006 | 1.12 (1.02-1.22) | 0.02 |
| D-dimer (µg/ml) | 2.32 (1.27-4.24) | 0.006 | 2.47 (1.33-4.59) | 0.004 |
| CRP (µg/ml) | 1.02 (0.97-1.06) | 0.47 | 1.02 (0.97-1.06) | 0.52 |
| IL-6 Q4 vs. Q123 | 2.49 (1.12-5.56) | 0.02 | 2.22 (0.93-5.30) | 0.07 |
| D-Dimer Q4 vs. Q123 | 3.52 (1.60-7.78) | 0.002 | 4.59 (1.98-10.67) | 0.0004 |
| CRP Q4 vs. Q123 | 1.25 (0.53-2.89) | 0.61 | 1.26 (0.50-3.17) | 0.62 |
| IL6 or D-dimer combination 1/2 vs. 0 | 3.59 (1.64-8.87) | 0.001 | 3.62 (1.58-8.28) | 0.002 |
